# Th1 promotes M1 polarization of intestinal macrophages to regulate colitis-related mucosal barrier damage

**DOI:** 10.18632/aging.204629

**Published:** 2023-04-04

**Authors:** Shuiliang Ruan, Liang Xu, Yongjia Sheng, Jin Wang, Xiaohong Zhou, Caiqun Zhang, Li Guo, Wenyan Li, Chenyang Han

**Affiliations:** 1 The Second Affiliated Hospital of Jiaxing University, Jiaxing 314001, Zhejiang, China; 2 Jiaxing University Master Degree Cultivation Base, Zhejiang Chinese Medical University, Hangzhou 310000, Zhejiang, China

**Keywords:** helper T cells, macrophages, M1 polarization, chronic colitis, mucosal barrier

## Abstract

This work aimed to investigate the role of helper T cell 1 (Th1) in chronic colitis and its immunoregulatory mechanism.

The proportions of Th1 and Th2, and the levels of related cytokines in tissues from patients with inflammatory bowel disease (IBD; ulcerative colitis+Crohn's disease, UC+CD) were detected. DSS was used to induce the mouse model of IBD; thereafter, Th1 cells were induced *in vitro* and amplified before they were injected intraperitoneally. Later, the changes in life state and body weight of mice were observed, the proportion of M1 macrophages in mucosal tissues and mucosal barrier damage were detected. After treatment with macrophage scavenging agent (Clodronate Liposomes, CLL), the influence of Th1 on IBD mice was observed. Then, the intestinal macrophages were co-cultured with Th1 *in vitro* to observe the influence of Th1 on the polarization of intestinal macrophages. Besides, cells were treated with the STAT3 inhibitor to further detect the macrophage polarization level. Intestinal macrophages were later co-cultured with intestinal epithelial cells to observe the degree of epithelial cell injury.

The Th1 proportions in intestinal tissues of UC and CD patients were higher than those in healthy subjects, but the difference in Th2 proportion was not significant. In the IBD mouse model, Th1 induced the M1 polarization of macrophages, aggravated the intestinal inflammatory response, and resulted in the increased mucosal barrier permeability. Pretreatment with CLL antagonized the effect of Th1 cells, reduced the intestinal tissue inflammatory response and mucosal barrier permeability.

## INTRODUCTION

Inflammatory bowel disease (IBD) is a kind of chronic intestinal inflammatory disease. As discovered from tissue detection of IBD patients, the number of intestinal macrophages apparently increases, At the same time, the intestinal macrophages have different subpopulations and phenotypes [1]. According to the different activation modes, macrophages can be classified as two types, including the classical activated macrophages (M1 type) and the alternative activated macrophages (M2 type) [2]. M1 macrophages can be activated by cytokines such as interferon-γ (IFN-γ) and tumor necrosis factor (TNF)-α [3]. Under the IBD status, M1 macrophages can secrete inflammatory cytokines IL-1α, IL-1β and TNF-β, which aggravate inflammatory response. Therefore, the M1 macrophages play an important pro-inflammatory role in IBD, which represent the major cell type inducing IBD progression [4].

At present, the immunoregulatory mechanism of M1 polarization of macrophages in IBD has not been completely revealed. Th cells are the helper T cells, which can also be divided into Th1 and Th2 cells. It is currently discovered that, Th1 cells are a kind of pro-inflammatory T cells, which exert mutual regulation with natural killer (NK) cells and macrophages [5]. Th2 cells are a kind of anti-inflammatory cells, which exert their effect via IL-10 [6]. In research on IBD, the change in Th cell proportions and the interaction with macrophages have not been completely illustrated yet. Th1 cells are the inflammatory cells with high expression of IFN-γ and TNF-α [7], therefore, this work analyzed the proportion of Th cells in tissues of IBD patients, so as to further reveal its regulatory relation with macrophages.

## MATERIALS AND METHODS

### Clinical sample selection and detection

From June 2019 to June 2021, 30 IBD patients admitted and treated in Gastroenterology Department of our hospital, including 15 with ulcerative colitis (UC) and 15 with Crohn’s disease (CD), were recruited into this study. Meanwhile, 15 healthy subjects receiving physical examination were enrolled for analysis. All the patients received enteroscopy and colonic tissues were obtained for biopsy. All patients provided the informed consent. The intestinal tissues were divided into two parts, one for the extraction of mononuclear cells to detect Th1 and Th2 cells, while the other one for protein extraction to detect Th1 cytokines IFN-γ, TNF-α and IL-2 as well as the Th2 cytokines IL-4, IL-10 and IL-13.

Extraction of tissue peripheral blood mononuclear cells (PBMCs) by Procell density gradient separation method [8]. After grinding, tissues were diluted with the RPMI-1640 medium and filtered with the 70-μm cell strainer. The homogenate was later centrifuged and resuspended with 5 ml medium. Then, 2.5 ml Procell separating medium (GE Healthcare, IL, USA) was added, followed by the addition of 70% Procell solution for 30-min centrifugation at 800 g/min. The white flocculent in the middle was PMBCs, after separation, cells were rinsed by HBSS solution twice.

Cytokines detected by ELISA assay: Intestinal tissues were rinsed by PBS twice, and grinded in liquid nitrogen until there was no granule. Then, 1 ml NP-40 cell lysis buffer (Beyotime Biotechnology Co., Ltd, Shanghai, China) was added to lyse on ice for 30 min to collect the supernatant protein solution. The levels of inflammation cytokines were detected in line with the ELISA kit (Jiancheng Institute of Biology, Nanjing, China), and the results were expressed as ng/ml.

Flow cytometry (FCM) was conducted to detect the proportions of Th1 and Th2 cells [9]. The separated PBMCs (including Th1 (CD4+IFN-γ+) and Th2 (CD4+IL-4+)) were adopted for detection. PBMCs were washed with the pre-chilled PBS twice, fixed with ethanol, and incubated with 10 μl antibodies for 20 min, including FITC-CD4 monoclonal antibody, PE-F4/80 monoclonal antibody, and APC-IL-4 monoclonal antibody (BD, MA, USA). After washing with PBS twice, cells were resuspended with 50 μl solution. Then, the samples were loaded for detection, and the results were expressed as %.

### Mouse model construction and intervention

The mouse model of chronic colitis was induced by dextran sulfate sodium (DSS) [10]. The female wild-type (WT) C57BL/6 mice weighing 21-25 g were used in the experiment after one-week of adaptive feeding. The animal experimental protocol was approved by the Ethics Committee of Jiaxing University Animal Experiment Center. All the animal experiments conformed to the guidelines of ethical norms and animal care. Mice were divided into Control, DSS and DSS+Th1 groups, with 10 mice in each group. Mice in Control group were raised routinely, those in DSS group were fed with DSS and used for the construction of IBD model, and those in DSS+Th1 group were given intraperitoneal injection of Th1 cells (thrice a day, 10^5^ cells) combined with DSS to construct the IBD model. The mouse model of chronic colitis was constructed through feeding with 2.5% DSS, to be specific, mice were given 2.5% DSS on days 1-5, 8-12, 15-19 and 22-26, whereas purified water on the remaining days. Four cycles were completed, the disease activity index (DAI) scores were rated, and mouse body weights were dynamically determined on days 1, 3 and 5 of each cycle. On day 29, mice were sacrificed for subsequent experiments. The distal ileum was separated till the rectum, and the intestinal tissues were rinsed by PBS solution (Sigma-Aldrich, MO, USA) thrice for subsequent detection.

Separation and induction of Th1 cells for experiment [11]. The spleens and lymph nodes of C57BL/6 mice were dissected, thereafter, cells in mouse spleens and lymph nodes were extracted according to the above-mentioned PBMC extraction method. Afterwards, CD4+ T cells were sorted with the CD4+ magnetic beads, and cell pellets were labeled with the anti-CD62L, anti-CD44, anti-CD25 and anti-CD4 fluorescent antibody complex. Each tissue from one mouse was treated with 100 μl antibody mixture. Later, the pellets were resuspended into the antibody mixture, and incubated at 4° C in dark for 15-30 min. After washing with 10 ml PBS, cells were centrifuged at 4° C and 475 g for 5 min. Thereafter, the CD4+CD25-CD62L+CD44- cell subpopulation was sorted, which represented the primary CD4+T cells. The collected cells were washed with complete RPMI medium. The well plates were coated with anti-CD3 and anti-CD28 antibodies ahead of time, then, 0.5 ml cell suspension (0.5×10^6^ cells) was added into each well of the 48-well plates, whereas 1 ml cell suspension (1×10^6^ cells) was added into each well of the 24-well plates. Th1 cells were induced with 15 ng/ml rmIL-12, 30 U/ml hIL-2 and 5000 ng/ml IL-4 continuously for 5-6 days, with medium exchange every two days. Finally, the Th1 cells (CD4+IFN-γ+) were obtained.

In mechanism research, WT mice were divided into Control, DSS, DSS+Th1 and DSS+Th1+CLL groups. CLL (Clodronate Liposome) can be used to scavenge macrophages [12], so as to exclude the influence of Th1 on macrophages. In the experiment, mice were given injection of CLL via the tail vein at the initial dose of 150 μL/mouse and 100 μL/mouse twice a day thereafter, for achieving the long-term depletion of macrophages. The other treatments were consistent with those mentioned above.

### Measurement of mouse body weight and DAI pathological score [13]

During the experimental cycle, the mouse body weight was dynamically measured. To be specific, the body weights were measured on days 1, 3 and 5 of each cycle, so as to calculate the ratio of mouse body weight to initial body weight (%). DAI score mainly evaluates the mouse pathological status. In line with the DAI scoring criteria, the mouse body weights, feces character and occult blood were evaluated on days 1, 3 and 5 of each cycle, and the scores were recorded. In the meantime, the mental status, activity, glossiness of hair, appetite and feces character of mice were observed every day. To determine the length of the colon, mice were sacrificed to dissect the colonic tissues for length measurement, and the results were expressed as cm. To rate the colonic morphology score, the colon was separated and the injury morphological score was rated according to specific standards.

### H&E

After the mice were sacrificed, the colonic tissues were dissected, embedded in paraffin and sliced into the 4-μm serial sections. Thereafter, the tissues were deparaffinized with xylene, dehydrated with ethanol at the gradient of 100%, 95% and 80%, washed with tap water for 2 min, and stained with xylene for 3 min. After washing with tap water for 2 min, the sections were treated with 1% hydrochloric acid alcohol for 2 s, washed with tap water for 2 min again, treated with 1% ammonium hydroxide for 20 s and with 0.5% eosin alcohol for 10 s. Thereafter, the sections were dehydrated with gradient alcohol, transparentized with xylene and mounted with neutral resin. Finally, the pathological changes of intestinal tissues were observed under the light microscope.

### Immunohistochemical (IHC) staining

The mouse intestinal tight junction (TJ) proteins (ZO-1 and Occludin) were stained. To be specific, the tissue sections were baked in the oven at 60° C, deparaffinized with xylene, and dehydrated with gradient alcohol, followed by antigen retrieval in the microwave at 98° C for 20 min. Later, the sections were incubated with 3% hydrogen peroxide at room temperature for 10 min to eliminate the endogenous peroxidase. After blocking with 2% bovine serum albumin (BSA) at room temperature for 30 min, the sections were incubated with monoclonal antibodies against ZO-1 and Occludin (Abcam, MA, USA; diluted with TBST at 1:500) at 4° C overnight. Thereafter, the sections were further incubated with IgG secondary antibody at 37° C for 15 min, and then with peroxidase-labeled streptomycin (Maixin Biotechnology Co., Ltd; Fuzhou, China) for another 15 min. Afterwards, the sections were washed with PBS thrice, with 5 min each time. Later, each section was dropwise added with freshly prepared DAB developing solution (DAKO, Denmark), counter-stained with hematoxylin and mounted. The negative control primary antibody was replaced by TBST. The Olympus-DP72 image collection system and the Olympus-BX51 upright microscope of the CRi Nauance multispectral imaging system were adopted for photographing and analysis.

### M1/M2 cells detected by FCM

The proportions of F4/80+CD11c+M1 cells and F4/80+CD206+M2 cells were detected by FCM. In brief, macrophages were treated with LPS/IFN-γ for 48 h, then cells were collected and washed with pre-chilled PBS twice, and fixed with ethanol. Later, cells were incubated with 10 μl antibodies (including FITC-CD11b, PE-F4/80, and APC-CD206 monoclonal antibodies; BD, MA, USA) for 20 min in dark. After washing with PBS twice, the cells were resuspended with 0.5 ml solution. The sample was later loaded for detection, and the results were expressed as %.

### Mucosal barrier permeability detected by FITC-D

Mice were given intragastric administration of fluorescein isothiocyanate-dextran (FITC-D) to detect its concentration and judge the changes in intestinal mucosal permeability. To be specific, after the completion of mouse intervention, the animals were subject to fasting for food and water at 4 h prior to sacrifice. Thereafter, the mice were given intragastric administration of FITC-D (MW=400, 60 mg FITC-D/100 g), and the serum was collected to detect the FITC-D concentration and fluorescence density of each sample with the fluorescence spectrophotometer.

### ELISA

In the animal experiment, the intestinal tissues were rinsed by PBS twice, and grinded with liquid nitrogen until there was no granule. Thereafter, 1 ml NP-40 cell lysate (Beyotime Biotechnology, Co., Ltd, Shanghai, China) was added for lysis on ice for 30 min, and then the supernatant protein solution was collected. Subsequently, inflammation cytokines were detected in line with the ELISA kit (Jiancheng Institute of Biology, Nanjing, China) instructions, and the results were expressed as ng/ml.

In the cell experiment, cells were inoculated into the 6-well plates. After co-culture, the medium was collected, and the separated medium was preserved at -80° C. After obtaining all the samples, they were centrifuged at 3000 r/min for 20 min and detected according to the ELISA kit (Jiancheng Institute of Biology, Nanjing, China) instructions. The standard curve method was utilized for calculation, and the results were expressed as ng/ml.

### Western-blot (WB assay)

The intestinal tissue proteins were extracted in line with ELISA assay. After protein quantification, the proteins were separated by electrophoresis and transferred onto the PVDF membrane. Later, the PVDF membrane was blocked with 5% defatted milk powder for 2 h, incubated with the TBST-diluted primary monoclonal antibodies (dilution, 1:300-1:500; Abcam, MA, USA) at 4° C overnight. Later, the membrane was further incubated with the HRP-labeled IgG secondary antibody (Abcam, MA, USA). The IgG was diluted with TBST at the volumetric ratio of 1:2000 for subsequent experiment. After incubation, the protein bands were detected with chemiluminiscence, and optical density (OD) was analyzed with the Image Pro-Plus 6.0 software, with GAPDH as the endogenous reference. The results were expressed as the OD ratio of target protein to endogenous protein.

### Co-culture of intestinal macrophages and Th1 cells

After the resuscitation of mouse intestinal macrophages (Wuhan Procell Biotechnology Co., Ltd, Wuhan, China), cells were cultured with the RPMI-1640+10% FBS complete medium under 37° C, 5% CO_2_ and saturated humidity conditions, and cells at logarithmic phase were utilized for experiment. Macrophages were divided into Control and Th1 groups. For cells in Th1 co-culture group, they were co-cultured at the ratio of Th1 cells: macrophages = 1:3 in the Transwell chambers.

In the STAT3 inhibitor experiment, macrophages were divided into Control, Th1 and Th1+Stattic groups. Stattic is the STAT3 inhibitor. In this study, macrophages were pre-treated with 20 μM Stattic for 6 h to block the STAT3 signal. Thereafter, macrophages were co-cultured with Th1 cells to observe the polarization level of macrophages.

### Mucosal epithelial cell assay

The monolayer formed by the mouse intestinal mucosal epithelial cells was used to mimic the *in-vitro* mucosal barrier. Epithelial cells were cultured in the Th1-treated intestinal macrophage culture, so as to observe the influence on the mucosal barrier permeability. Cells were divided into Control and M1 groups. Among them, cells in Control group were the non-intervened epithelial cells, whereas those in M1 group were epithelial cells cultured in the M1 macrophage medium after Th1 treatment.

### Determination of transepithelial electrical resistance (TEER)

The mouse intestinal mucosal epithelial cells were inoculated into the Transwell chambers at a density of 4×10^5^ cells/mL. 200 μL was added into each well, later, 600 μL 1640 medium was added into the basement membrane. The experiment was initiated when cells formed the tight cell layer, and TEER was detected by the Millcell resistance machine after co-culture with macrophage medium for 24 h. The TEER measurement process was conducted at the constant temperature of 37° C, and three points at different directions of each Transwell chamber were selected for repeated measurements in triplicate. The resistance value was expressed as ohm/cm2 (Ω/cm2). The standard TEER value = (measured value - blank control measured value)/0.33 cm^2^.

### Mucosal epithelial cell permeability detected by FITC-D

The mouse intestinal mucosal epithelial cells were inoculated into the Transwell chamber and co-incubate with intestinal macrophages for 24 h. After careful washing with Hanks solution thrice, 1 mg/mL FITC-D was added from the top of the Transwell chamber, while 0.6 mL blank HBSS was added into the basement membrane to incubate for 1 h at 37° C. Thereafter, solution on the basement membrane side was collected, and fluorescence intensity was measured with the fluorescence spectrophotometer (excitation wavelength 490 nm, emission wavelength 520 nm). The FITC-D concentration was calculated according to the FITC-D standard curve. The FITC-D penetration rate (%/h/cm^2^) = (FITC-D fluorescence intensity on the basement membrane side/FITC-D fluorescence intensity on the top of the Chamber)/1h/0.33cm^2^×100%.

### Apoptosis detected by FCM

The cell apoptosis level was detected. To be specific, epithelial cells were co-cultured with the macrophage medium for 24 h, then the suspension cells and adherent cells were collected. After washing with pre-chilled PBS twice, cells were fixed with methanol, then Annexin V-FITC and PI in the cell apoptosis kit (BD, MA, USA) were incubated with 15 μl antibody for 20 min in dark. After washing twice with PBS, cells were resuspended with 50 μl solution. The sample was later loaded for detection, and the results were expressed as %.

### TUNEL staining

The mouse intestinal mucosal epithelial cells were co-incubated with the intestinal macrophage medium for 24 h. After washing with PBS twice, cells were incubated with protease K for 30 min in the incubator. Then, cells were washed with PBS, and cell membrane was broken by adding the membrane breaking solution. The TdT and dUTP in TUNEL kit were mixed at a ratio of 1:9, and the mixture was dropwise added onto the sections to further incubate for 2 h. After nuclear counter-staining, the TUNEL-positive cell number was observed under the microscope.

### Statistical analysis

All the measurement data were expressed as (±s), analyzed and processed with the SPSS 17.0 software. After homogeneity test of variance, two independent sample t-test was applied for data analysis between two groups, while one-way ANOVA was adopted for data comparison among three groups or more, and the LSD method was applied for pairwise comparison between groups thereafter. The above-mentioned tests were two-sided, and P<0.05 stood for statistical significance.

### Data availability statement

The data that support the findings of this study are available from the corresponding author upon reasonable request.

## RESULTS

### Proportions of Th1/Th2 cells and the expression of cytokines in tissues of IBD patients

**Figure 1:**
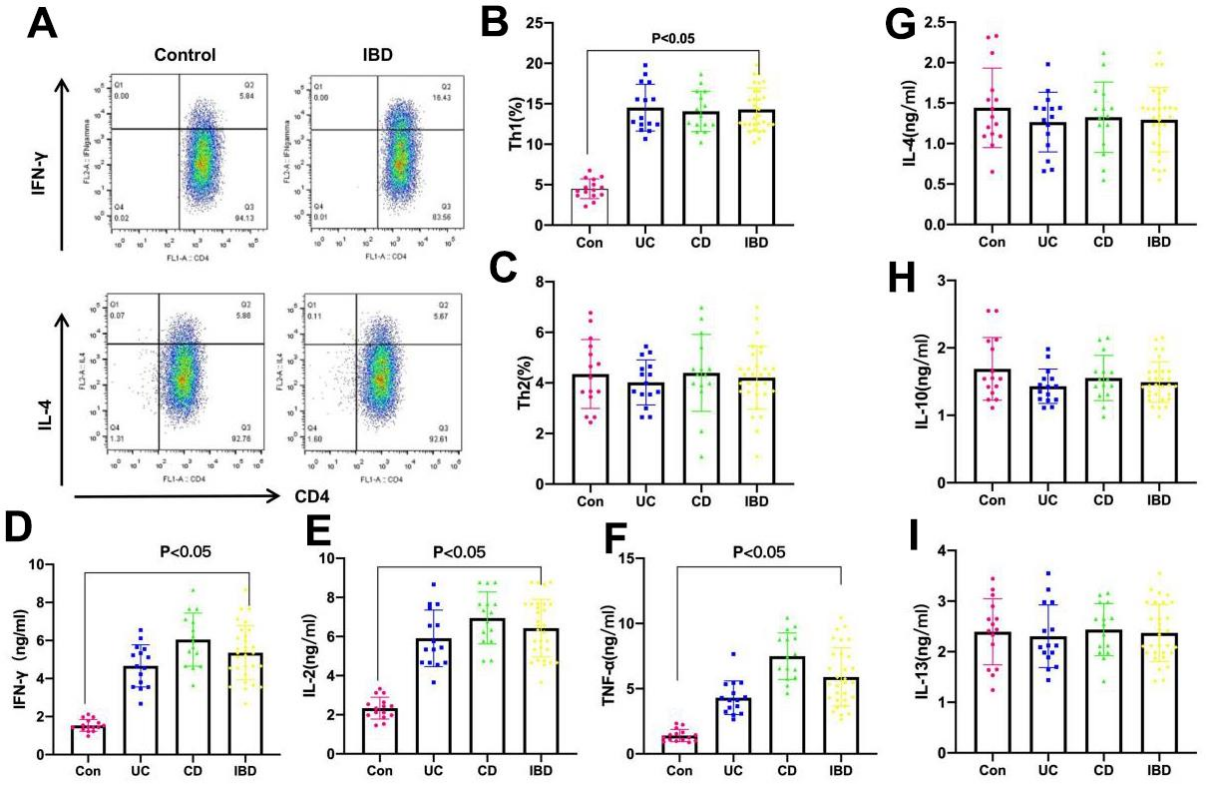
**Proportions of Th1 and Th2 cells and expression of cytokines in tissues of IBD patients.** (**A**–**C**) Proportions of Th1 and Th2 were detected by FCM. As a result, the proportions of Th1 cells in CD and UC groups were significantly higher than that in Control group, and that was significantly different between IBD (UC+CD) and Control groups, while Th2 cell proportion did not exhibit any significant difference. The proportion of Th1 cells in IBD increased. (**D**–**F**) The levels of Th1 cell marker factors IFN-γ, IL-2 and TNF-α. The levels of IFN-γ, IL-2 and TNF-α in IBD (UC+CD) were evidently higher than those in Control group, and those in CD patients were higher than those in UC patients, with significant difference. (**G**–**I**) The levels of Th2 cell marker factors IL-4, IL-10 and IL-13. The levels of IL-4, IL-10 and IL-13 in IBD (UC+CD) group were not significantly different compared with those in Control group.

The proportions of Th1 and Th2 cells in tissues of IBD patients were detected, as a result, the proportion of Th1 cells in CD and UC patients significantly increased compared with Control group, while Th2 cell did not exhibit any significant difference, suggesting the increased proportion of Th1 cells in IBD (Figure 1A–1C). IFN-γ, IL-2 and TNF-α are the characteristic factors of Th1 cells, and their levels in UC and CD patients markedly increased compared with Control group, and those in CD patients were higher than those in UC patients (Figure 1D–1F). IL-4, IL-10 and IL-13 are the characteristic factors of Th2 cells, and their expression levels were not significantly different between Control and UC or CD groups (Figure 1G–1I).

### Effect of Th1 cells on mice with chronic colitis

**Figure 2:**
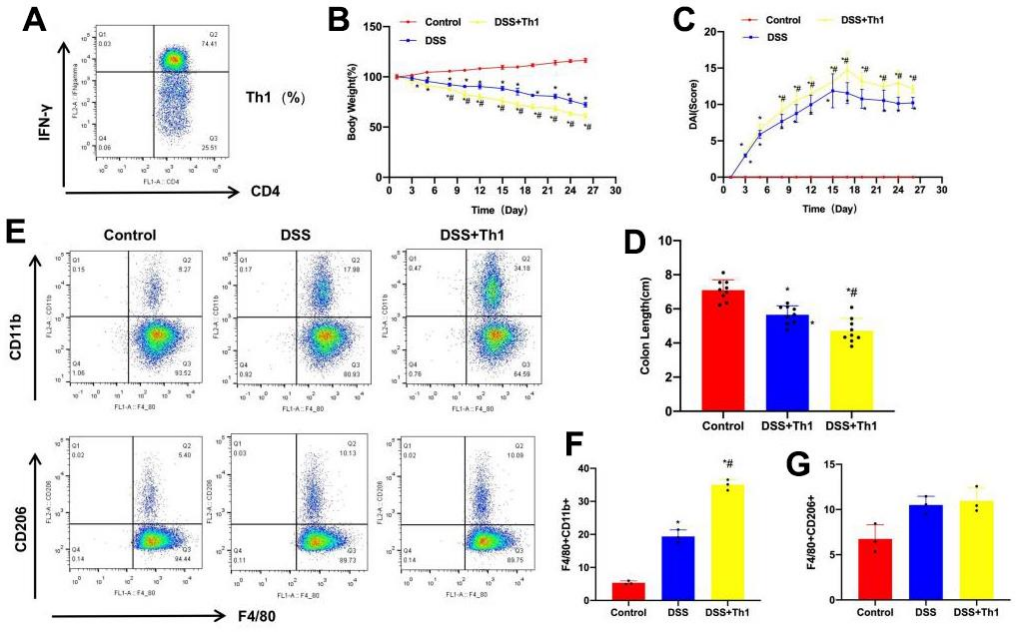
**Th1 promoted the aggravation of mouse colitis and M1 polarization of tissue macrophages.** (**A**) Th1 cells were highly pure after induction and amplification, which were used for subsequent experiments. (**B**) Dynamic detection of mouse body weight (n=10). The mouse body weight apparently declined after DSS administration, and the difference was significant compared with Control group, while Th1 injection further reduced mouse body weight. ^*^P<0.05 compared with Control group, ^#^P<0.05 compared with DSS group. (**C**) DAI scores of mice (n=10). DAI scores in DSS group significantly increased compared with Control group. In dynamic detection, the DAI scores increased with time, Th1 increased the DAI score, and the difference was significant compared with DSS group. ^*^P<0.05 compared with Control group, ^#^P<0.05 compared with DSS group. (**D**) Mouse intestinal length (n=10). Relative to Control, the intestinal tissue length of DSS mice was shortened, Th1 injection aggravated mouse intestinal injury, and further reduced the intestinal tissue length. ^*^P<0.05 compared with Control group, ^#^P<0.05 compared with DSS group. (**E**–**G**) Proportions of M1/M2 macrophages (n=10). The proportion of M1 cells in DSS increased, and the difference was significant compared with Control group. The proportion of M1 cells in DSS+Th1 group further increased, higher than that in DSS group. There was no significant difference in M2 cell proportion. ^*^P<0.05 compared with Control group, ^#^P<0.05 compared with DSS group.

Th1 cells were cultured *in vitro* and amplified for subsequent experiments (Figure 2A). According to our results, DSS administration led to colitis in mice. Besides, compared with Control group, the body weight of DSS mice apparently declined, the DAI score increased, and the intestinal tissue length of mice was shortened. Th1 injection aggravated mouse intestinal injury, the body weight of DSS+Th1 mice further decreased, DAI score increased, and the differences were significant compared with DSS group, meanwhile, the intestinal tissue length was further shortened (Figure 2B–2D). PBMCs were separated from tissues, as a result, the proportion of M1 macrophages in DSS group increased, and the difference was significant compared with Control group. The proportion of M1 macrophages in DSS+Th1 group further increased, higher than that of DSS group. By contrast, the proportion of M2 macrophages was up-regulated, but there was no significant difference in M2 cell proportion between DSS and DSS+Th1 groups (Figure 2E–2G).

**Figure 3:**
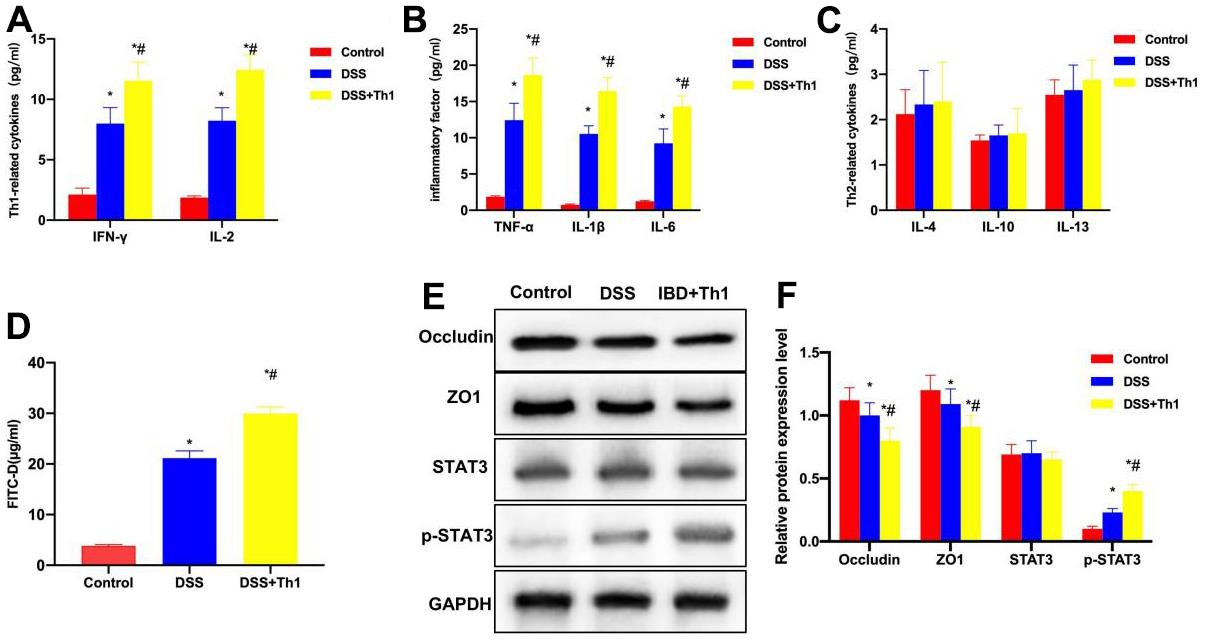
**Th1 aggravated intestinal inflammation and reduced the TJ protein levels.** (**A**) Th1 cell characteristic factors (n=10). The expression of IFN-γ and IL-2 in DSS significantly increased, while that in DSS+Th1 group further increased relative to DSS group, and the difference was significant compared with DSS group. ^*^P<0.05 compared with Control group, ^#^P<0.05 compared with DSS group. (**B**) Detection of inflammation cytokines (n=10). The levels of TNF-α, IL-6 and IL-1β in DSS group significantly increased, higher than those in Control group. The inflammatory factor levels in DSS+Th1 group further increased, and Th1 improved their tissue levels. ^*^P<0.05 compared with Control group, ^#^P<0.05 compared with DSS group. (**C**) Th2 cell characteristic factors (n=10). The levels of IL-4, IL-10 and IL-13 were not significantly different among Control, DSS and DSS+Th1 groups. (**D**) FITC-D (n=10). The FITC-D penetration rate in DSS group significantly increased, while that in DSS+Th1 group further increased relative to DSS group, and the mucosal permeability was apparently enhanced. ^*^P<0.05 compared with Control group, ^#^P<0.05 compared with DSS group. (**E**, **F**) Protein detection results (n=5). DSS induced the down-regulation of TJ proteins ZO1 and Occuldin, and their expression further decreased in DSS+Th1 group. At the same time, the STAT3 phosphorylation level increased. ^*^P<0.05 compared with Control group, ^#^P<0.05 compared with DSS group.

As discovered from the detection of Th1 cell characteristic factors, the expression of IFN-γ and IL-2 in DSS group significantly increased, while that in DSS+Th1 group was further up-regulated compared with DSS group, and the difference was significant (Figure 3A). According to inflammatory factor detection, the levels of TNF-α, IL-6 and IL-1β in DSS group significantly increased, higher than those of Control group. Moreover, the inflammatory factor levels in DSS+Th1 group further increased, and Th1 cells increased the TNF-α, IL-6 and IL-1β levels in tissues (Figure 3B). It was discovered from the detection of Th2 cell characteristic factors that, the levels of IL-4, IL-10 and IL-13 were not significantly different among Control, DSS and DSS+Th1 groups (Figure 3C). When detecting the mouse intestinal mucosal permeability, the FITC-D penetration rate in DSS group significantly increased, while that in DSS+Th1 group further increased relative to DSS group, and the mucosal permeability apparently increased (Figure 3D). In protein detection, DSS led to the down-regulation of TJ proteins ZO1 and Occuldin, while their expression was further down-regulated in DSS+Th1 group, and the STAT3 phosphorylation level increased at the same time (Figure 3E, 3F).

**Figure 4:**
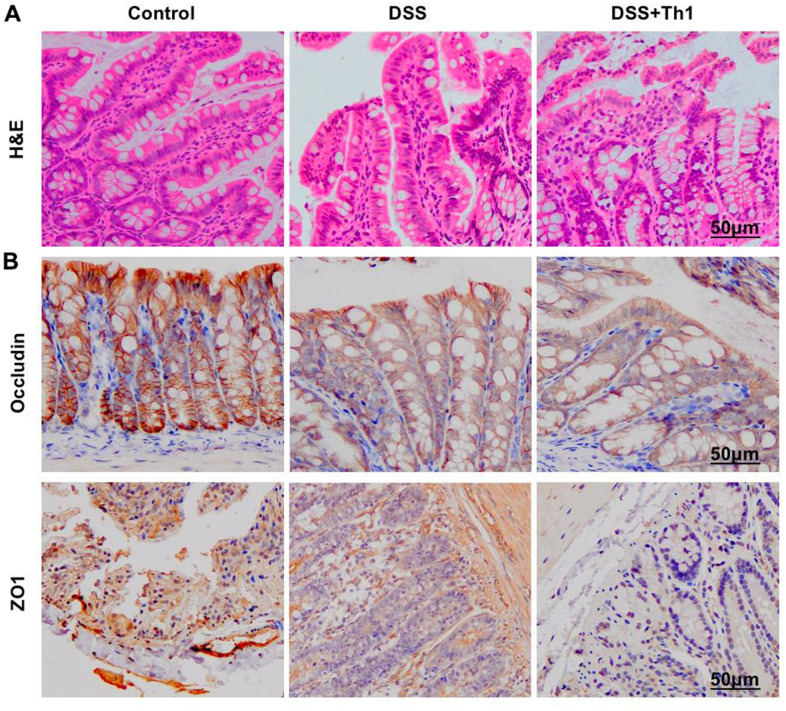
**Effect of Th1 on the intestinal tissue pathology of mice.** (**A**) H&E (n=5). Apparent inflammatory injury occurred in intestinal tissue of DSS group, along with evident goblet cell injury, while Th1 aggravated the injury and further destroyed the intestinal mucosal structure. (**B**) IHC (n=5). In TJ protein staining, the levels of ZO1 and Occludin significantly decreased, and Th1 further suppressed the expression of TJ proteins, and the difference was significant compared with DSS group.

H&E staining revealed obvious inflammatory injury in intestinal tissues, along with apparent goblet cell injury. Th1 aggravated the injury and further broke the intestinal mucosal structure (Figure 4A). In TJ protein staining, the levels of ZO1 and Occludin significantly decreased, while Th1 further suppressed the expression of TJ proteins, with significant difference compared with DSS group (Figure 4B).

### Scavenging macrophages resisted the effect of Th1 cells

**Figure 5:**
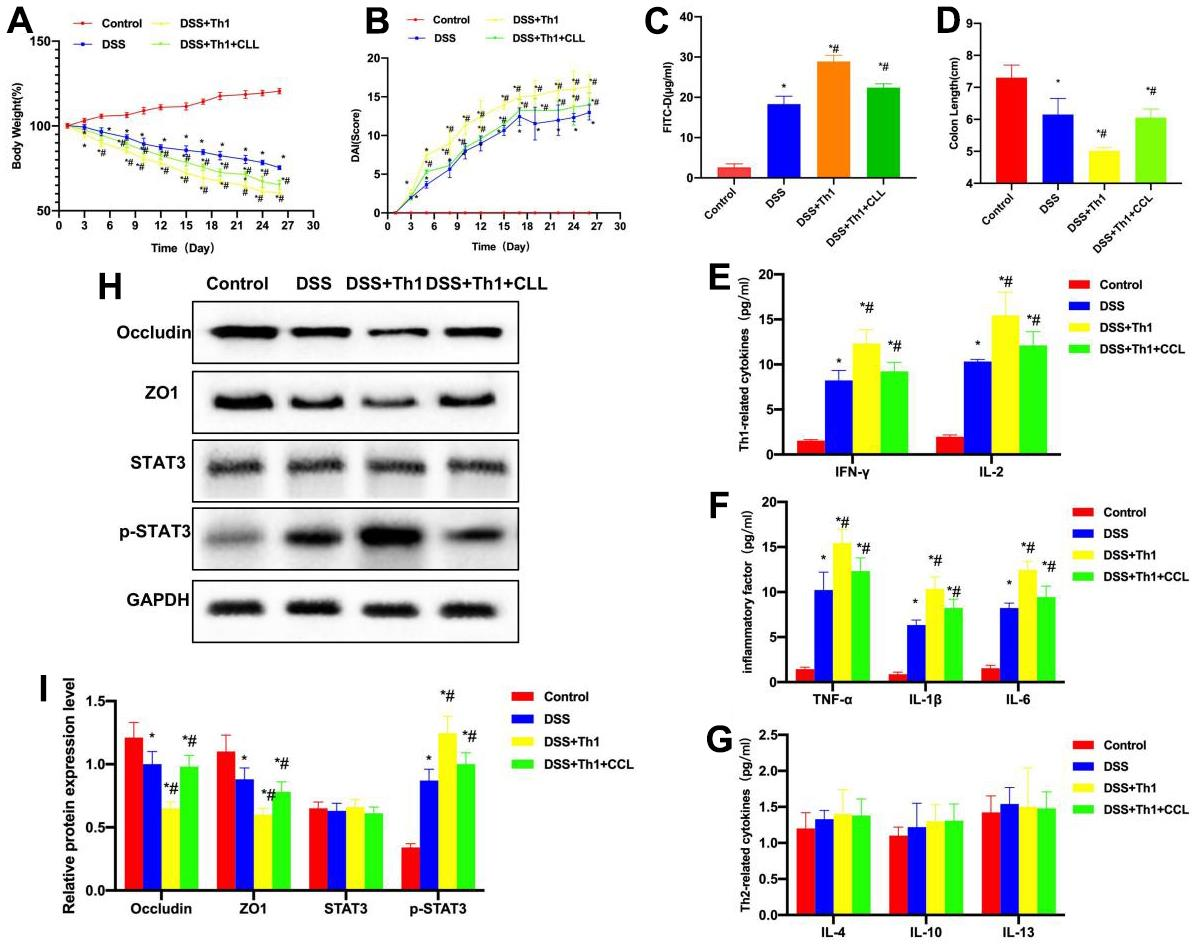
**Macrophage scavenging alleviates the Th1-induced chronic colitis in mice.** (**A**) Dynamic detection of mouse body weight (n=10). After CCL was used to scavenge macrophages, it resisted the effect of Th1 cells. According to mouse body weight detection, the reduction of body weight in DSS+Th1+CCL group was markedly alleviated relative to DSS+Th1 group, and the difference was significant. ^*^P<0.05 compared with Control group, ^#^P<0.05 compared with DSS group. (**B**) Mouse DAI score (n=10). The DAI scores of DSS+Th1+CCL group markedly decreased relative to DSS+Th1 group. ^*^P<0.05 compared with Control group, ^#^P<0.05 compared with DSS group. (**C**) FITC-D (n=10). In the detection of mouse intestinal mucosal permeability, the FITC-D level of DSS+Th1+CCL group markedly decreased relative to DSS+Th1 group, and the permeability decreased. ^*^P<0.05 compared with Control group, ^#^P<0.05 compared with DSS group. (**D**) Mouse intestinal length (n=10). The intestinal length of DSS+Th1+CCL group was extended compared with DSS+Th1 group. ^*^P<0.05 compared with Control group, ^#^P<0.05 compared with DSS group. (**E**, **F**) Detection of Th1 cell markers and inflammation cytokines (n=10). The levels of IL-2 and IFN-γ as well as inflammation cytokines TNF-α, IL-1β and IL-6 were changed, and the cytokine levels in DSS+Th1+CCL group were evidently reduced relative to DSS+Th1 group, and the difference was of statistical significance. ^*^P<0.05 compared with Control group, ^#^P<0.05 compared with DSS group. (**G**) Th2 cells-related factors (n=10). There was no significant difference in the IL-4, IL-10 and IL-13 levels. (**H**, **I**) Protein detection results (n=5). CCL improved the TJP levels, the levels of ZO1 and Occludin proteins in DSS+Th1+CCL group were markedly higher than those in DSS+Th1 group, while the level of p-STAT3 decreased. ^*^P<0.05 compared with Control group, ^#^P<0.05 compared with DSS group.

After CCL injection, macrophages in the tissues were scavenged to prevent the effect of macrophages. According to our results, after CCL was used to scavenge macrophages, it resisted the effect of Th1 cells. As discovered by mouse body weight detection, the body weight loss in DSS+Th1+CCL group significantly decreased compared with DSS+Th1 group, and the difference was significant (Figure 5A). DAI scores also revealed that, the DAI scores of DSS+Th1+CCL group were markedly reduced relative to DSS+Th1 group (Figure 5B). In mouse intestinal mucosal permeability detection, the FITC-D level in DSS+Th1+CCL group significantly decreased relative to DSS+Th1 group, along with reduced permeability (Figure 5C). According to detection of intestinal tissue length, the length of DSS+Th1+CCL group was extended relative to DSS+Th1 group (Figure 5D). In mouse intestinal tissues, the expression levels of Th1 cell markers IL-2 and IFN-γ and the inflammation cytokines TNF-α, IL-1β and IL-6 were changed, and cytokines in DSS+Th1+CCL group were significantly down-regulated relative to DSS+Th1 group, and the differences were significant (Figure 5E, 5F). The levels of Th2 cell markers IL-4, IL-10 and IL-13 did not exhibit any significant difference (Figure 5G). Protein detection suggested that, CCL improved the TJP levels, ZO1 and Occludin levels in DSS+Th1+CCL group significantly increased relative to DSS+Th1 group, while p-STAT3 level decreased (Figure 5H, 5I).

**Figure 6:**
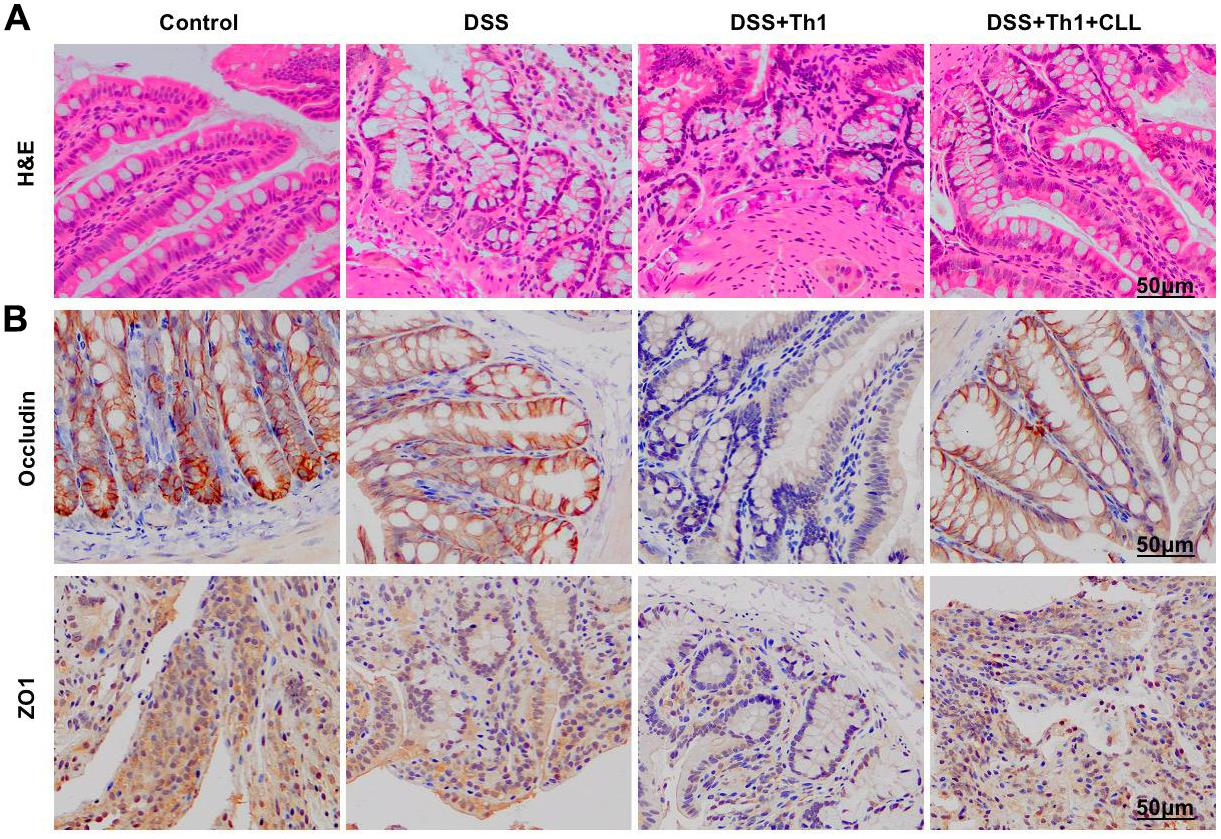
**Changes of intestinal histopathology after the scavenging of mouse macrophages.** (**A**) H&E (n=5). In DSS+Th1+CCL group, the tissue exhibited obvious inflammation and edema, which were markedly alleviated relative to DSS+Th1 group. (**B**) IHC (n=5). The levels of ZO1 and Occludin proteins in DSS+Th1+CCL group were markedly higher than those in DSS+Th1 group.

In histopathological detection, DSS+Th1+CCL group displayed obvious inflammation and edema in H&E staining, but the severity was significantly alleviated compared with DSS+Th1 group (Figure 6A). In IHC staining, TJP levels were significantly changed, with the levels of ZO1 and Occludin proteins in DSS+Th1+CCL group being significantly higher than those in DSS+Th1 group (Figure 6B).

### Th1 promoted the M1 polarization of intestinal macrophages *in vitro*

**Figure 7:**
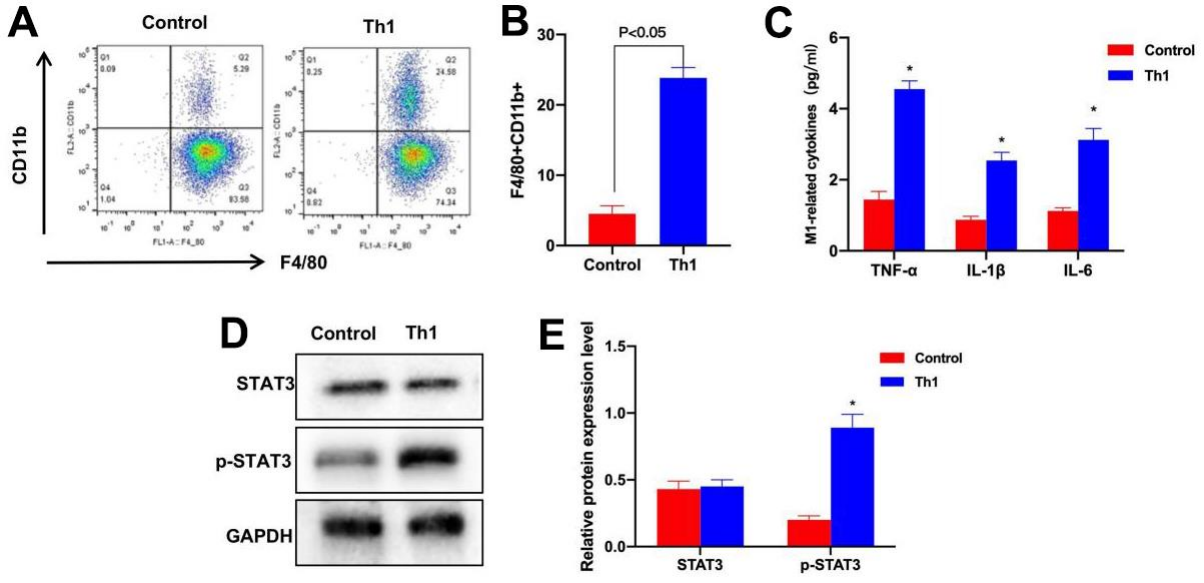
**Th1 cells promoted M1 polarization of intestinal macrophages.** (**A**, **B**) FCM (n=3). The proportion of F4/80+CD11b+ cells in Th1 cells evidently increased, and the difference was significant compared with Control group, *P<0.05. (**C**) ELISA (n=3). The levels of TNF-α, IL-6 and IL-1β markedly increased, and the differences were significant compared with Control group, *P<0.05. (**D**, **E**) Protein detection results (n=3). The p-STAT3 level in Th1 cells was markedly up-regulated, and the difference was significant compared with Control group, *P<0.05.

Th1 cells were co-cultured with intestinal macrophages, as a result, the M1 polarization of macrophages in Th1 cells was obvious, and the proportion of F4/80+CD11b+ cells remarkably increased (Figure 7A, 7B). Meanwhile, the levels of M1 cell markers TNF-α, IL-6 and IL-1β evidently increased (Figure 7C). The expression levels in Th1 cells were higher than those in Control group, and protein detection also revealed the evidently up-regulated p-STAT3 level (Figure 7D, 7E).

### Suppressing STAT3 signaling resisted the effect of Th1 cells

**Figure 8:**
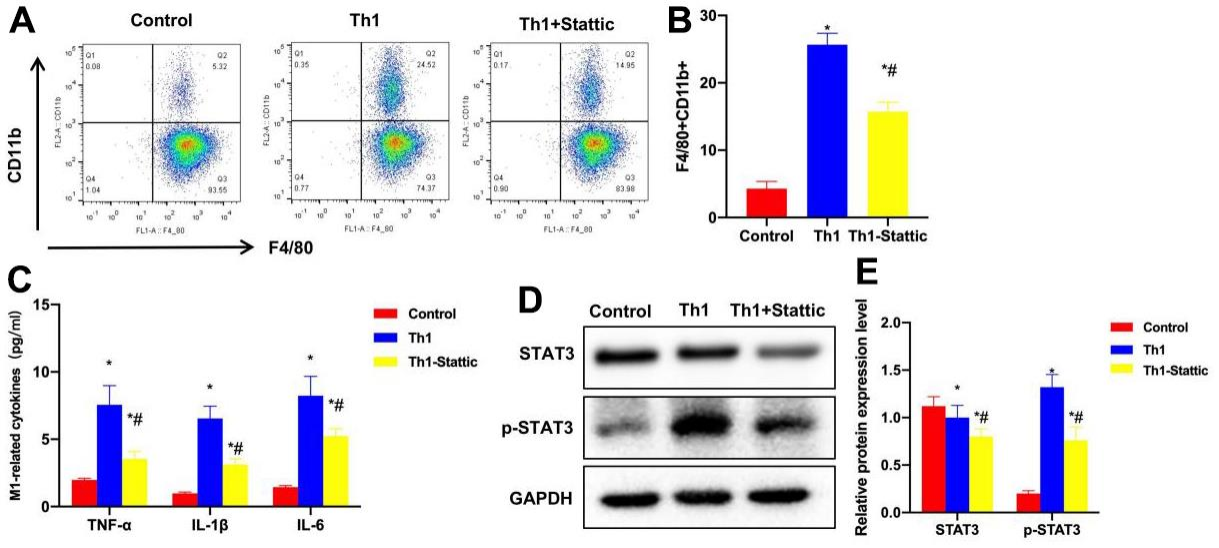
**Suppressing STAT3 signaling antagonizes the effect of Th1 cells.** (**A**, **B**) FCM (n=3). Stattic treatment antagonized Th1 cells, the proportion of M1 cells significantly decreased, and that of Th1+Stattic group was remarkably lower than that of Th1 group. ^*^P<0.05 compared with Control group, ^#^P<0.05 relative to Th1 group. (**C**) ELISA (n=3). The levels of M1 cell markers TNF-α, IL-6 and IL-1β significantly decreased in Th1+Stattic group, lower than those of Th1 group. ^*^P<0.05 compared with Control group, ^#^P<0.05 relative to Th1 group. (**D**, **E**) Protein detection (n=3). The expression levels of STAT3 and p-STAT3 in Th1+Stattic group significantly decreased. ^*^P<0.05 compared with Control group, ^#^P<0.05 relative to Th1 group.

Stattic is the STAT3 phosphorylation inhibitor. In this study, Stattic treatment antagonized the effect of Th1 cells, the proportion of M1 cells significantly decreased, and that in Th1+Stattic group markedly reduced relative to Th1 cells (Figure 8A, 8B). The M1 cell markers TNF-α, IL-6 and IL-1β were apparently down-regulated in Th1+Stattic group relative to Th1 group (Figure 7C). Protein detection also discovered the evidently decreased levels of STAT3 and p-STAT3 (Figure 7D, 7E).

### The Th1-induced M1 cells induced epithelial cell injury

**Figure 9:**
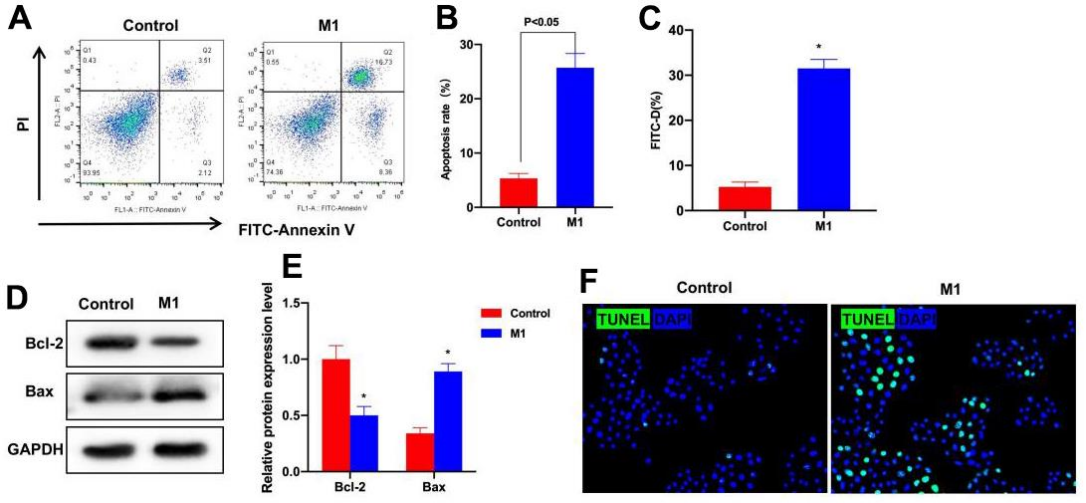
**Th1 induced M1 cells can induce epithelial cell injury.** (**A**, **B**) FCM was conducted to detect cell apoptosis (n=3). The cell apoptosis rate significantly increased in M1 group, higher than that of Control group. (**C**) FITC-D was adopted to detect the cell barrier permeability (n=3). M1 damaged the cell layer, increased the FITC-D level and enhanced the permeability. ^*^P<0.05 compared with Control group. (**D**, **E**) Protein detection (n=3). The expression of Bax increased, and that of Bcl-2 decreased, and the difference was significant compared with Control group. ^*^P<0.05 (**F**) TUNEL staining (n=3). The positive cell number in M1 group significantly increased, higher than that of Control group.

The induced M1 cell medium was used to culture the intestinal epithelial cells, as a result, the cell apoptosis rate in M1 group significantly increased, higher than that of Control group (Figure 9A, 9B). FITC-D permeability detection discovered that, M1 injured the cell layer, increased the FITC-D level and enhanced the permeability (Figure 9C). Protein detection also suggested that the expression of apoptosis-related Bax increased, that of Bcl-2 decreased, and the difference was significant compared with Control group (Figure 9D, 9E). TUNEL staining results also revealed that the number of positive cells in M1 group significantly increased, higher than that of Control group (Figure 9F).

## DISCUSSION

Helper T cells are the important immune cells mediating inflammatory response [14], which can be divided into Th1 and Th2 cells that exert different immune effects [15, 16]. Th1 cells are mainly dependent on IL-2 and IFN-γ [17], induce the macrophage-dependent immune response, enhance the toxic effect of natural killer (NK) cells, and mediate hypersensitivity reaction to cause tissue injury [18]. Th2 cells mainly secrete IL-4 and IL-10, which can promote the IgE-mediated hypersensitivity reaction and humoral immune response [19]. Th2 cells mainly function to exert the anti-inflammatory effect, and can antagonize bacterial and viral infections. Th2 and Th1 cells mutually regulate each other, Th2 can also down-regulate the Th1 cells-mediated inflammatory response, and restrict the induced tissue injury [20]. Therefore, regulation of Th1/Th2 cells is of great importance for inflammatory disease. Inflammatory bowel disease (IBD) mainly includes ulcerative colitis and Crohn’s disease [21]. The pathogenic mechanism of IBD is related to the comprehensive consequence of genetic, environmental and immune factors [22]. Typically, macrophages have a particularly important role in IBD, which are the important phagocytes and antigen-presenting cells in the body [23]. Under the action of different inducing factors, macrophages can be classified as M1 and M2 polarized types according to the phenotypes and secreted cytokines. The macrophage polarized phenotype in IBD patients is changed, which is dominated by M1 type [24]. Under the action of the IFN-γ and TNF-α classical activation pathways factors, macrophages can be polarized into M1 type, while the M1 macrophages can efficiently present antigens and secrete the pro-inflammatory cytokines at a high level [25]. During the progression of IBD, massive M1 macrophages represent a major cause leading to mucosal injury.

At present, the interaction between Th1 cell proportion and macrophages in IBD research has not been completely illustrated. In this study, the Th1/Th2 cells and marker cytokines in IBD patients and healthy human intestinal tissues were detected. As a result, the proportions of Th1 cells in CD and UC patients significantly increased, but there was no difference in Th2 cells, suggesting the dominance of Th1 cells in IBD. At the same time, the levels of IL-2, TNF-α and IFN-γ were up-regulated, but those of Th2 cell markers [26] IL-10, IL-13 and IL-4 were not significantly changed. To explore the role and mechanism of Th1 in IBD, the induced Th1 cells were injected. According to our results, Th1 induced the increased proportion of M1 cells in tissues, but did not affect M2 cells. M1 cells are the pro-inflammatory macrophages, which secrete a large amount of inflammation cytokines like IL-6, IL-1β and TNF-α. Meanwhile, Th1 aggravated mouse intestinal inflammation, further reduced the body weight, increased the DAI score, and shortened the intestinal tissue length. Microstructure detection suggested that, Th1 aggravated the intestinal tissue inflammatory response, edema, inflammation and intestinal villous destruction. In our opinion, Th1 promoted the M1 polarization of macrophages, aggravated the intestinal tissue inflammatory response and thus destroyed the mucosal barrier. To prove that Th1 exerted the effect via interacting with macrophages, the macrophage scavenging agent CLL was used. As a result, after applying CLL, the macrophages in tissues were scavenged, indicating that CLL antagonized the effect of Th1 cells, alleviated the intestinal tissue injury, in particular, the villous-like structure was relatively complete. At the same time, after the administration of CCL, the inflammatory factor levels in tissues were down-regulated, while the expression of TJP was up-regulated. ZO1 and Occludin are the linker proteins maintaining mucosal barrier, and their expression levels reflected the mucosal barrier integrity. Besides, CLL increased the expression of ZO1 and Occludin, and reduced the serum FITC-D level, suggesting the decreased mucosal barrier permeability and improved integrity. In the meantime, the mouse body weight loss was alleviated, and the DAI score was reduced. In the *in-vitro* experiments, Th1 cells were co-cultured with intestinal macrophages, and it was found that Th1 cells promoted the M1 polarization of intestinal macrophages, significantly increased the proportion of F4/80+CD11b+ cells, meanwhile, the levels of M1 cell markers IL-6, IL-1β and TNF-α also increased. STAT3 is the important regulatory signal of M1 polarization [27, 28]. To verify that Th1 cells exert their effect via STAT3, cells were pretreated with the STAT3 phosphorylation inhibitor. As a result, inhibition of STAT3 signal antagonized the effect of Th1 cells, and the M1 polarization of intestinal macrophages was suppressed *in vitro*. Finally, the Th1-induced macrophage medium was used to culture epithelial cells, according to our results, the Th1-induced macrophages also exhibited inflammatory injury, increased epithelial cell apoptosis rate, and improved inflammatory factor levels. Therefore, it was determined that Th1 cells mainly induced M1 polarization of macrophages to exert their effect, while Th1-STAT3-M1 macrophages induced IBD progression, thus further aggravating intestinal injury.

## CONCLUSIONS

The proportion of Th1 cells in the intestinal tissues of IBD patients increases, while that of Th2 cells is not significantly changed. Th1 induces the M1 polarization of intestinal macrophages to induce local inflammatory response, aggravate intestinal inflammation and mucosal barrier injury in IBD patients. At the same time, the interaction between macrophages and Th1 cells is the major mode by which Th1 exerts its effect on IBD, since scavenging macrophages apparently antagonizes the effect of Th1.
